# Gender and Gender Role Differences in Self- and Other-Estimates of Multiple Intelligences

**DOI:** 10.1080/00224545.2012.754397

**Published:** 2013-05-13

**Authors:** Agata Szymanowicz, Adrian Furnham

**Affiliations:** YSC, London; University College London

**Keywords:** gender, gender role, intelligence, personality, self-assessed intelligence, self-estimates of intelligence

## Abstract

This study examined participant gender and gender role differences in estimates of multiple intelligences for self, partner, and various hypothetical, stereotypical, and counter-stereotypical target persons. A general population sample of 261 British participants completed one of four questionnaires that required them to estimate their own and others’ multiple intelligences and personality traits. Males estimated their general IQ slightly, but mathematic IQ significantly higher than females, who rated their social and emotional intelligence higher than males. Masculine individuals awarded themselves somewhat higher verbal and practical IQ scores than did female participants. Both participant gender and gender role differences in IQ estimates were found, with gender effects stronger in cognitive and gender role than in “personal” ability estimates. There was a significant effect of gender role on hypothetical persons’ intelligence evaluations, with masculine targets receiving significantly higher intelligence estimates compared to feminine targets. More intelligent hypothetical figures were judged as more masculine and less feminine than less intelligent ones.

THIS STUDY SETS OUT to examine whether the hubris/humility effect in the estimation of self and other intelligence scores is primarily related to gender or gender role. A consistent gender difference effect has been noted in studies of self-estimated intelligence where females, socialized in “humility,” consistently give lower scores than males who have been socialized in “hubris” and who give higher self-estimates of general intelligence (Beloff, 1992; Furnham & Shagabutdinova, 2012; Kaufman, 2012; Szymanowicz & Furnham, 2011a, 2011b). Interestingly, recent German studies have shown that neither actual measured intelligence nor gender-stereotypical parental perceptions totally explain boys’ stronger confidence in their intelligence (Steinmayr & Spinath, 2009).

## Self Estimates Intelligence

Studies of gender differences in self-estimated intelligence are now done in over 30 countries and five continents (Freud & Kasten, 2012). What is interesting is that in nearly every study there are similar, consistent, and significant differences between males and females in self-estimates of overall (general) intelligence, with males giving higher scores to themselves. There are, however, cultural differences in level with, for instance, Africans and Americans awarding themselves higher scores (over one-and-a-half standard deviations above the mean) while the Chinese and Japanese tend to give more modest scores, around half a standard deviation above the mean. Clearly there are inevitable cultural differences in the concept of intelligence, which informs self and other estimates (Furnham, 2001).

The overall aim of this study is to examine how the gender and gender role of participants influences the perception of their own intelligence (and multiple intelligences), that of their partner as well as hypothetical others who are either described in “gender consistent” stereotypical ways or inconsistently. Whilst there is a large and growing literature on gender differences in estimated intelligence, there is far less work on the role of gender role on intelligence estimates. Further, this study advances work in this field by examining intelligence estimates of males and females in stereotypic and non-stereotypic jobs to see the role of a person's occupation in the estimation of their intelligence. Finally, the study looks at personality trait ratings of hypothetical males and females described as having very high IQs (in the top 2% of the population) to determine how participants gender and gender role influences their perception, particularly of very intelligent women (Szymanowicz & Furnham, 2011a, 2011b).

## Multiple Intelligences

Part of the theoretical background of the study is derived from Gardner's (1983) theory of multiple intelligence which asserts that intelligence, defined as “the ability to solve problems or to create products that are valued within one or more cultural settings” (p. 11) is made up of distinct, unrelated, multiple abilities. The original theory rejected the notion of general intelligence and described seven “intelligences” which are unrelated to one another. A large body of research done in many different countries indicates that males consistently tend to estimate their intelligence, especially mathematical and spatial abilities, significantly higher than females (Furnham, 2001; Swami & Furnham, 2010)

Studies on gender differences in intelligence ratings of others show the same pattern as self-ratings but with less significant “discriminatory” trends. People's ratings of their mothers’, grandmothers’ and daughters’ intelligence tend to be significantly lower than those of their fathers’, grandfathers’ and sons’, respectively (Bennet, 1996; Furnham et al., 2001; Furnham & Rawles, 1995; Neto & Furnham, 2011). Moreover, most studies looking at the estimated intelligence of others have been based on real people (relatives, famous people) rather than on experimentally manipulated, hypothetical “target” people. There have, however, been a number of studies that have looked at the estimation of the intelligence of others (Carney, Colvin & Hall, 2007; Swami & Furnham, 2012).

## Gender Role

This study will investigate the role of gender as well as gender differences on these estimates. The Bem Sex Role Inventory (BSRI) is one of the most widely used gender measures. Although there have been criticisms of the scale and other scales that have been developed, it remains extensively used in psychometric studies (Colley, Mulhern, Maltby & Wood, 2009: Rammsayer & Troche, 2007). Individuals are classified as having one of four gender roles: masculine, feminine, androgynous, or undifferentiated (Bem, 1974). The androgynous individual is defined as a female or male who has a high degree of both feminine (expressive) and masculine (instrumental) traits. A feminine individual is high on feminine (expressive) traits and low on masculine (instrumental) traits. A masculine individual is high on instrumental traits and low on expressive traits. An undifferentiated person is low on both feminine and masculine traits. The measure has been subjected to considerable psychometric assessment (Holt & Ellis, 1998) and used successfully with participants similar to those in this study where the alpha for masculinity was .86 and that for femininity was .77 (Storek & Furnham, 2012).

According to gender schema theory set out by Bem (1981), people utilize culture-specific definitions of masculinity and femininity as standards against which they categorize, evaluate and perceive their own behavior and that of others. Gender-typed people (masculine males; feminine females) often engage in gender schematic processing while non-gender-typed people (both/neither sex typed: androgynous/undifferentiated) tend to be more gender aschematic using gender less as an organizing perceptual dimension (Schmitt & Millard, 1988). The theory has attracted considerable attention (Best & Thomas, 2004; Eagly, Wood & Diekman, 2000; Spence & Buckner, 2000). Thus, we predict gender schematic individuals may attribute higher intelligence to males than females, while gender aschematic individuals would not differentiate between the two.

Two early European studies are relevant. Using the Personal Attributes Questionnaire (Spence, Helmreich & Strapp, 1975) to measure masculinity and femininity Furnham, Clark and Bailey (1999) in Great Britain, found sex differences more powerful determinants of self-estimates of multiple intelligences rather than gender role (or their interaction). Rammstedt and Rammsayer (2002) tested German participants using the BSRI and a measure of self- estimated multiple intelligence. They found that gender differences were significant on two of their four factors and that gender role was not significant on any, but they found a strong interaction on the mathematical-logical intelligence factor where high masculine males had much higher estimates than feminine males. They concluded that they found “direct evidence for the notion that in male, but not in female individuals, self-estimates of specific aspects of intelligence are markedly influenced by gender role role” (p. 380). Both studies used student samples and only self-estimates while the present study set out to systematically examine the effects of participant gender and gender role on self-estimates of “multiple intelligences” as well as estimates of partner and perceived intelligence of hypothetical gender-stereotypical and non stereotypical characters.

Various self-estimated intelligence studies have shown that logical reasoning, as well as mathematical and spatial intelligence is considered a masculine sphere, whereas interpersonal and emotional “intelligence” or skills are more often regarded as feminine domains (Beloff, 1992; Bennett, 2000; Rammstedt & Rammsayer, 2000). For instance, Bennett (2000) reported that mathematical, spatial, and kinaesthetic intelligences were judged as more masculine, while personal, musical, and verbal intelligences were judged as more feminine. Furnham (2001) argued that the concept of intelligence is *male normative*, which accounts for the systematic and universal gender differences in self estimates. Other studies show that gender differences favoring males in self-estimated mathematical abilities occur in all cultures as well as in children and early adolescents, despite the fact that there are either no gender differences or else girls outperform boys (Hyde et al., 1990; Rammstedt & Rammsayer, 2001). In addition, girls typically receive better grades than boys, which could be expected to be the main source of information on related abilities for school children.

Gender differences in self-estimates and other-estimates of intelligence seem partly influenced by gender-oriented stereotypes. Intelligence, as conventionally defined, in general may be perceived as a more masculine than feminine feature and thus create a certain degree of conflict between intelligence and femininity. If this were the case, such inconsistence would be noted more by gender-typed people—those with higher femininity or masculinity scores (Bem, 1981, 1993; Markus et al., 1982)—who are more aware of the gender role dimension and more attuned to cultural definitions of masculinity and femininity (Bem, 1982). Such individuals would be expected to differentiate more clearly between males and females in their intelligence estimations. Moreover, because gender-schemas are incorporated into their self-concept (Bem, 1981; Markus et al., 1982), gender-typed people would also view themselves in accordance with cultural gender stereotypes and would also be more motivated to comply with them. Thus, lower self-estimates of intelligence would be expected in feminine individuals, whereas the opposite would apply to masculine individuals. This will be tested in this study.

In addition, perceived masculinity or femininity of a hypothetical person or imaginary target should affect intelligence ratings made by gender-schematics. Consequently, people may rate an imaginary target as being more masculine if he or she is assumed to have a higher IQ and vice-versa. On the other hand, self-estimates of intelligence of men and women who are not gender-typed should be similar, as should ratings of other males’ and females’ intelligence.

This study was done in Great Britain with a majority of British-born participants. It is well established that cultural factors do influence gender-based attitudes as well as self- and other-estimates of intelligence (Best & Williams, 1994). For instance, gender attitudes have been shown to be closely related to Hofstede's (2001) cultural dimensions on masculinity/femininity and power distance. On both these dimensions, the United States and the United Kingdom (as well as other English speaking and predominant Anglo Saxon cultures like Australia, Canada and New Zealand) are very similar. The same is true for the now extensive literature on cross-cultural differences in self-estimated or self-assessed intelligence (Furnham, 2001; Szymanowicz, & Furnham, 2011a, 2011b). This suggests that it would be acceptable to use United States based supporting research used to derive hypotheses in this study, but that similar results may not be found in very different cultures.

## This Study

This study examined the relative effect of masculinity and femininity: Is it that “those who adopt feminine cultural stereotypes tend to underestimate their own intelligence” (Furnham et al., 1999, p. 255), or rather is it masculinity that makes people overestimate their abilities?

It tests the following hypotheses:

## Self and partner's estimates of intelligence:

*Hypothesis 1a (H1a)*: Males will estimate their mathematical and spatial intelligence higher than females.

*H1b*: Females will estimate their social and emotional intelligence higher than males.

*H1c*: Masculinity will be positively related to self-estimated general, mathematical, spatial and practical intelligence and negatively to social and emotional intelligence.

*H1d*: Femininity will be positively related to verbal, social and emotional intelligence and negatively to general, mathematical, spatial and practical intelligence.

*H1e*: Males will estimate their partner's mathematical and spatial intelligence lower than their own and social and emotional intelligence higher than their own; the opposite will be true of females.

## Vignettes’ intelligence estimates:

*H2a*: There will be an effect of target's gender role on intelligence estimates: masculine cues will receive higher general and multiple intelligences’ estimates comparing to feminine cues.

*H2b*: Masculine vignettes’ will receive higher estimates of mathematical than verbal IQ; Feminine vignettes will receive higher estimates of verbal than mathematical IQ.

*H2c*: A highly intelligent hypothetical female will be rated as more masculine and less feminine than a less intelligent female.

*H2d*: A highly intelligent hypothetical female cue will be rated as less likeable, less attractive, less feminine and more likely to be single than a less intelligent female hypothetical person.

*H2e*: A highly intelligent male cue will be rated as more likeable, more attractive, more masculine and less likely to be single than less intelligent male cue.

We did not make any specific predictions for those individuals classified as androgynous or undifferentiated.

## Method

### Participants

In all, 377 participants aged 18 to 77 years (*M* = 38.35, *SD* = 14.5 years) took part: 167 of them were females (mean age 38.96 years; *SD* = 14.32), 181 males (mean age 36.92, *SD* = 14.80), and 29 had missing data and were therefore removed prior to data analysis. Those under 21 and over 70 were removed from the analyses, as well as those with same sex partners. The remaining age distribution was normal. The final analyses were done on 261 individuals (134 female, 127 male). They were all members of the general population, recruited on commuter trains by the first author. Seventy percent of the participants were born in the UK, and 79% had English as a first language; while nearly 80% were resident in the UK. Seventy-six percent of them described themselves as White, 20% as British other ethnic background (including Black, African, Asian, European and mixed). There were no significant first language or nationality/ethnicity differences in any of the measures. In all 31% of the participants were married, 29% had a partner, 27% were single and 4% were divorced.

### Measures

There were four versions of each questionnaire. Participants completed a four-page questionnaire that consisted of five parts, namely:

*Intelligence estimates.* Participants were asked to rate themselves and their opposite sex partner (preferably current or former in they did not have a current partner) on overall IQ and six specific types of intelligence using a pictorial bell-curve of standardized IQ scores. The multiple intelligences used were: verbal, mathematical, and spatial (Gardner, 1983), social and practical (Sternberg, Conway, Ketron & Bernstein, 1981) and emotional (Goleman, 1996). There was a short definition for each: For instance, emotional intelligence was described as “the ability to understand and manage your own and others emotions.” These were chosen on the basis of previous research, which suggested that these abilities would yield the biggest gender differences (Furnham, 2001). Each label had a brief description of the nature of that particular intelligence (i.e. “Spatial Intelligence: the ability to find your way around the environment and form mental images”).

*Vignettes I—Intelligence ratings.* Participants were given descriptions of four people—two males and two females—indicating their occupation, hobbies, interests and four personality traits. One male and one female were more traditionally masculine, the other male and other female were more feminine. Three of the personality traits used in each description were taken from the masculinity and femininity scales of either Personal Attributes Questionnaire (PAQ) or Bem Sex Role Inventory (BSRI), the fourth—from BSRI neutral scale (Bem, 1981; Spence et al., 1975). Targets’ hobbies and interests were chosen to reflect cultural stereotypes of males and females; two of them were sex-typed, one was neutral. A pilot study testing 20 people confirmed the validity of the descriptions—i.e., that relevant descriptions were judged as more typically masculine or feminine. Three additional descriptions of famous British people/celebrities (Jade Goody, David Beckham and Margaret Thatcher), constructed in the same way as the main vignettes, were also given and served as distracters. Participants were asked to estimate general, verbal and mathematical intelligences of the hypothetical seven persons. Estimates of the three celebrity targets were not scored.

*Vignettes II—Person traits rating.* Participants read a short description of one person. In 50% of the cases the target was female (Anna), whereas in the other 50% it was male (James). These two groups were further split into two subgroups, where information about the target's membership in MENSA (the high IQ society) or the “Walking Association” was given. Both texts were equivalent regarding their structure and length and resulted in four description versions (sex × IQ information). There were thus four different parts to this questionnaire, and participants completed only one, which they received on a random basis. Here is an example:

James Gordon lives in Birmingham. He graduated in International Studies with French from the local university. His first job was in a bank and now he works for a publishing company. James is interested in music and films, enjoys playing tennis, going out to the movies and restaurants and meeting with his friends in a local pub. He is a long-term member of IML Walking Association—an international organisation that promotes walking as a worthwhile recreation and organizes international walking events.

Participants were asked to rate the target on 10 items. Six of them were masculine and feminine characteristics from PAQ and BSRI inventories. Two items came from the PAQ's Male-Valued (M), and two from the Female-Valued (F) scale. These scales consist of personality traits stereotypically believed to differentiate between the genders but considered to be socially desirable in both (Robinson et al., 1991). The remaining two items came from PAQ's Sex-Specific (M-F) scale, which is comprised of personality traits “stereotypically believed to differentiate between sexes and considered to be more desirable in one sex than the other” (p. 597). The six items were as follows: competitive (M); independent (M); warm to others (F); understanding (F); dominant (masculine, M-F); needs approval (feminine, M-F). Additionally, participants estimated how likeable, attractive, feminine/masculine and likely to be single/have a partner this person was. All 10 items were presented in a 5-point bipolar format (e.g. 1: *very cold in relations with others*, 5: *very warm in relations with others*), and participants chose the number that they thought best described the target.

*IQ questions and demographic information.* Participants provided some demographic information (gender, age, country of birth, ethnicity, mother tongue, marital status, their own and their parents’ education, number of brothers and sisters and how religious they are) and answered the following questions (in yes/no format): Have you ever taken an intelligence test? Do you believe they measure intelligence fairly well? Do you believe intelligence is primarily inherited?

*Bem Sex Role Inventory.* Participants completed Bem Sex Role Inventory (BSRI; Bem, 1974; 1981) which assesses individuals’ masculinity and femininity based on self-reported possession of masculine and feminine, which are socially desirable personality characteristics. It consists of 60 items—20 masculine, 20 feminine, and 20 neutral fillers. Based on the masculinity and femininity scores obtained BSRI enables assignment of subjects into four gender role groups: masculine, feminine, androgynous and undifferentiated. Masculine and feminine individuals can be further divided into gender- typed and cross-gender typed, depending on the congruence or incongruence of gender and gender role. In this study the alpha for Masculinity was .78 and that of Femininity was .75. It is often recommended that the measure is subject to factor analysis to confirm that the traits are similarly grouped in different cultures: this was done, but the ratio of items to subjects could lead to factor instability. Because of the satisfactory nature of the alphas, the original scoring was retained for the analysis.

### Procedure

Testing took place on inter-city trains and participants were recruited while making their journeys. We have used this recruitment method to get a more representative sample of the population in many other studies very successfully. In all 447 people were approached out of which 32 (7%) refused to participate, 12 returned blank and 26 were incomplete questionnaires which could not be scored (e.g. both BSRI and information about gender were missing). Ethical permission was sought and received for the study. Participants were thanked and debriefed but not renumerated for their time

## Results

### BSRI and Intelligence Estimates: Missing Values and Outliers

Participants were classified into four gender role groups using a median split procedure (Robinson et al., 1991): undifferentiated (female = 42/28%; male = 46/29%), feminine (female = 56/37%; male = 16/10%), masculine (female = 17/11%; male = 56/35%) and androgynous (female = 35/23%; male = 42/26%). 38 participants either did not fill in BSRI or their assignment into a gender role group was impossible due to too many missing values. As previous studies showed (Furnham, 2001) gender differences can sometimes be caused by a small number of outliers on self-estimated intelligence ratings; hence they were removed (list-wise). A two-way (gender by gender-role) ANOVA or chi-square (where appropriate) on various biographical factors (age, education, ethnicity, marital status) failed to yield any significant differences.

### Gender and Gender Role Differences in Self-Estimates of Intelligence

Two MANOVAs with six multiple intelligences as dependent variables and gender and gender role as independent variables were computed for self- and partner-estimates (see Table 1). All alpha values in the tables are two tailed. The same analyses were run co-varying participants’ age, education and other demographic variables but this did not change the results. Given the high correlations between general and multiple intelligences, general IQ estimates were not entered in the MANOVAs but analyzed separately through ANOVAs. This analysis allowed for the testing of *H1a* to *H1e*.

**TABLE 1.** ANOVA (General Intelligence) and MANOVA (Multiple Intelligences) Results for Self-Estimates of Multiple Intelligences

	Gender		Gender role (GO)				F level		
Intelligence	*F*	*M*	Und	Fem	Mas	And	G	GR	G × GR
General	114.4	117.2	115.1	113.9	117.6	116.7	3.26	1.16	0.13
Verbal	111.7	115.5	111.6	112.1	116.3	114.3	2.15	3.09*	5.61***
Maths	107.5	115.0	108.9	109.1	116.4	110.4	10.61***	1.22	1.01
Spatial	109.7	116.6	111.0	112.1	114.9	114.4	18.79***	1.00	0.18
Social	118.1	116.7	112.5	119.3	119.1	119.4	2.23	6.32***	1.06
Emotional	117.1	111.7	112.0	115.9	112.0	118.1	16.74***	4.75**	0.76
Practical	111.5	113.1	108.6	111.0	116.9	113.1	0.02	5.62***	0.05

*p <.05, **p < .01, ***p < .001 (all two tailed).

Und: undifferentiated, Fem: feminine, Mas: masculine, And: androgynous, G: gender, GR: gender role; *N_F_* = 134; *N_M_* = 127.

Males estimated their general IQ slightly higher than females, and masculine individuals awarded themselves somewhat higher scores than did feminine. However, neither of these differences were significant (*F*(1, 260) = 1.76, *p* = .10; *F*(1.260) = 1.03, *p* = .41, respectively). On four out of six multiple intelligences males estimated their IQ higher than females; on mathematical and spatial intelligence significantly so, which supported *H1a*. Females had higher self-estimates than males in social and emotional intelligence (only the latter difference being significant) thus *H1b* was confirmed.

Masculine participants estimated their verbal (*p* < .05) and practical intelligence (*p* < .01) significantly higher than feminine and undifferentiated individuals and their emotional intelligence significantly lower than feminine (*p* < .05) and androgynous (*p* < .01). The undifferentiated group provided the lowest self-estimates, rating their social intelligence lower than that of all other groups (*p* < .001). They also estimated their emotional IQ lower than feminine (*p* < .05) and androgynous (*p* < .001) and their practical intelligence lower than masculine (*p* < .001) and androgynous (*p* < .05) participants. There was also an interaction between sex and gender role on self-estimated verbal intelligence. Within all but masculine gender role groups, males awarded themselves higher scores than females. Masculine females, however, estimated their verbal intelligence not only higher than masculine males but higher than men and women from all other gender role groups.

Table 2 displays two sets of effect sizes *d* (corrected for bias) for gender differences in intelligence self-estimates: without and with the effect of gender role co-varied. Two series of one-way ANOVA's (with and without gender role co-varied) were run to obtain *F* values for computation of the effect sizes. However, in order to ensure better comparability with the results of MANOVA presented here, cases that were excluded from MANOVA as multivariate outliers were also excluded from these ANOVA's.

**TABLE 2.** Effect Sizes for Gender Differences in Self-Estimated Multiple Intelligences Without and With Covarying Gender Role

	Gender differences in intelligence self-estimates	
IQ	*d*	*d* (gender role covaried)
General	0.29	0.24
Verbal	0.31	0.28
Math	0.57	0.55
Spatial	0.56	0.54
Social	−0.06	−0.19
Emotional	−0.45	−0.60
Practical	0.23	0.08

Effect sizes computed using *F* values; corrected for bias.

Negative *d* values indicate higher females’ self-estimates.

It can be seen that, consistently with the MANOVA's results, co-varying gender role did not have a substantial effect on the effect sizes of gender differences in general, verbal (small effects), mathematical and spatial (medium-size effect) intelligences. It did, however, change *d*s for the remaining abilities. For social intelligence co-varying gender role increased the effect size from non-existent to small whereas on practical intelligence the opposite was true. In case of emotional intelligence co-varying gender role increased the effect size by 0.15 standard deviations, which resulted in the largest gender difference of all analysed abilities.

Estimates of partners’ intelligence revealed gender differences in mathematical (*F*(1,260) = 12.78, *p* < .001) and spatial (*F*(1,260) = 4.93, *p* < .05) intelligence. Women gave higher scores to their partners (*M_Partner_* = 116.4, *M_Self_* = 112.8) than did men (*M_Partner_* = 109.3, *M_Self_* = 109.3). This is what was predicted by *H1e*; however, other predictions referring to social and emotional intelligences were not supported.

To assess the relative importance of masculinity and femininity on self-estimates of more traditional, cognitive, and “soft” abilities and compare these effects to that of gender all self and partner's IQ estimates were regressed on the three above mentioned variables. The results are presented in Table 3.

**TABLE 3.** Regressions of Self-Estimates of General and Multiple Intelligences on Sex, Masculinity, and Femininity

	Gender			Masculinity			Femininity				
Intelligence	*r*	*β*	*t*	*r*	*β*	*t*	*r*	*β*	*t*	*F*	*R*^2^
General	.17**	0.10	1.40	.19**	0.16	2.32*	−.09	−0.05	−0.74	4.20**	0.05
Verbal	.15**	0.07	1.02	.19**	0.18	2.83**	−.06	−0.07	−1.16	5.06**	0.05
Maths	.30**	0.26	4.10***	.15**	0.07	1.10	−.12*	−0.05	−0.85	9.95***	0.09
Spatial	.27**	0.26	4.12***	.25**	0.12	2.02*	−.04	0.05	0.78	10.97***	0.10
Social	−.14*	−0.22	−3.56***	.24**	0.31	5.26***	.19**	0.13	2.27*	15.89***	0.14
Emotional	−.22*	−0.22	−3.44***	.06	0.12	1.96	.30**	0.25	4.20***	15.41***	0.14
Practical	.11*	0.01	0.16	.23**	0.22	3.62***	.05	0.04	0.66	5.64***	0.05

*p < .05, **p < .01, ***p < .001 (all two tailed).

For gender, negative *t*-values indicate higher females’ self-estimates.

As seen, in the case of self-estimates of multiple intelligences on which gender role groups differed significantly, the differences were due to the effect of masculinity. Moreover, this effect was always positive and in case of general, verbal, social and practical IQs exceeded that of gender. Such effect of masculinity mostly contradicted what had been hypothesised (*H1b*). Femininity turned out to be a much less influential predictor.

Three exploratory factor analyses of six multiple intelligences—unrotated as well as using orthogonal and oblique rotation—were then carried out. Rotated factors yielded a clearer two factor solution with verbal, mathematical, spatial and practical intelligence loading onto the first and emotional and social intelligence onto second factor.

Factor analytic results for partners’ estimates were similar, though practical intelligence loaded highly on both factors. The first factor, formed by verbal, logical, spatial, and practical abilities was labelled “cognitive,” whereas social and emotional intelligences formed a “personal” factor. Factor scores were obtained by adding together the scores of the items loading on that factor. Linear regression showed that only intelligences loading onto cognitive factor were significant predictors of general IQ self-estimates. For partner's estimates, general IQ was predicted by verbal, mathematical/logical, spatial, and emotional intelligence.

Next, a gender × gender role MANOVA on the two factors was run. There were main effects of both independent variables on both factors but no interactions (see Table 4). Compared to gender role, the effect of gender was relatively stronger on the cognitive factor whereas on the personal factor the opposite was true. On the cognitive factor, masculine participants rated themselves significantly higher than undifferentiated (*p* < .001), feminine (*p* < .01) and androgynous (*p* < .05) whereas the undifferentiated group thought they were less intelligent than their androgynous counterparts (*p* < .05). On the personal factor, undifferentiated participants granted themselves lower scores than feminine (*p* < .05), masculine (*p* < .01) and androgynous (*p* < .001) individuals. Again, multiple regression revealed stronger effect of masculinity than femininity; however, for personal factor, femininity was a better predictor than sex, which was not significant.

**TABLE 4.** MANOVA Results for Gender and Gender Role Effect on Self-Estimated Intelligence on Two Factors

	Gender		Gender role (GO)				*F* level		
Factor	*F*	*M*	Und	Fem	Mas	And	Gen	GO	Ge × GO
Cognitive	110.09	114.98	109.95	111.21	116.12	113.03	10.59***	3.83**	0.41
Personal	114.27	112.12	109.99	113.71	113.90	115.60	5.12*	5.53***	0.45
*N*	134	132	74	60	65	67			

*p < .05, ***p < .001 (all two tailed).

### Differences Between Self- and Partner-Ratings

In order to further explore the relationship between self- and partner-estimates, the difference between these scores was computed by deducting the estimated partner's score from self-estimated score, such that positive results indicated greater self-estimates and negative results indicated greater scores awarded to one's partner. As can be seen (see Table 5), women on average estimated their overall intelligence lower than that of their partners, while men gave themselves higher scores than they attributed to their partners (*M_F_* = −3.29, *M_M_* = 2.26; *χ^2^* = 16.33, *p* < .001). Feminine females on average judged their general IQ to be 5 points lower than their partner's score but in the case of masculine females the difference was 6 points in the opposite direction. In the male sub-sample only undifferentiated participants estimated their partners’ general IQ score slightly above their own (*M* = −1.09). A two-way ANOVA with gender and gender role as independent variables yielded significant result (*F*(2,259) = 2.91, *p* < .01), but none of the main effects were significant (although gender role approached significance: *F*(2,259) = 2.53, *p* = .058).

**TABLE 5.** ANOVAs (General Intelligence) and MANOVAs (Multiple Intelligences) Results for Gender and Gender Role Effect on Difference Between IQ Estimates for Self and Partner

	Gender		Gender role (GO)				*F* level	
Intelligence	*F*	*M*	Und	Fem	Mas	And	Gen	GO
General	−3.29	2.26	−1.98	−3.94	5.24	0.14	2.35	2.53
Verbal	−2.23	0.62	−3.01	−4.54	4.74	0.41	0.14	5.14**
Maths	−9.57	5.48	−1.29	−7.89	6.87	−5.08	30.04***	1.11
Spatial	−4.56	7.07	2.96	−3.91	5.69	0.90	18.25***	0.94
Social	3.63	−2.49	−3.14	3.68	−0.59	3.10	8.37**	1.89
Emotional	8.83	−2.38	4.04	3.40	−3.87	8.63	24.56***	3.45*
Practical	−0.90	3.42	1.00	−1.47	5.51	0.25	3.17	0.75

*p < .05, **p < M, ***p < .001 (all two tailed).

*N_F_* = 115; *N_M_* = 130.

The largest gender differences between self and partner's estimates were found on mathematical, spatial, and emotional intelligences. Males awarded themselves higher and females lower scores than they gave to their partners on the first two abilities and the discrepancy was 15.1 and 11.6 points, respectively. The opposite pattern was found for emotional and, to a smaller extent, social intelligence, where the respective differences in favor of women were 11.21 and 6.12 points. Effects regarding gender roles were observed on verbal and emotional IQs. Even though women estimated their verbal abilities lower than that of their partners’, there were clear discrepancies between masculine and feminine women. The first group placed themselves 10 points higher and the second 5 points lower than their partners.

The differences between respective gender role groups in the male sub-sample were in the same direction (negative for feminine and positive for masculine males) but the discrepancy was smaller: 6.8 points. On emotional intelligence all females, regardless of their gender role, placed themselves higher than their partners, but androgynous women perceived this difference as largest (13 points). A similar effect was found for androgynous males who were the only gender role group within the male sub- sample who thought they were emotionally more intelligent than their partners. Relative effect of masculinity and femininity in comparison to gender was assessed by running a set of regressions.

Masculinity was an important predictor of difference between self and partner's estimates on verbal and social intelligence, whereas femininity affected these perceptions in regards to general and mathematical intelligence. However, these effects were opposite: self-enhancement for masculinity and self-degradation for femininity.

### Vignettes’ Intelligence Ratings

The analysis of responses to the vignettes allowed for the testing of *H2a* to *H2e*. MANOVAs of four targets’ ratings for two questionnaire versions for descriptions (see method section) were carried out to assess whether paired descriptions (within the same gender role) were equivalent. Results showed that two masculine descriptions were rated differently (*p* < .001) (masculine description 1: *M_Male_* = 120.34, *M_Female_* = 119.28; Masculine description 2: *M_Male_* = 126.21, *M_Female_* = 124.13). However, further analysis revealed that there were no differences between male and female targets described with the same vignette (*t_1_* = 1.17, *df* = 357; *t_2_* = 2.29, *df* = 364, *t_crit_* = 2.326) so it was decided to average the two descriptions and conduct one analysis for the whole sample. Three repeated measures ANOVAs (excluding outliers) for general, verbal and mathematical IQ with gender and gender role as within-subjects factors were run. Means and standard deviations for all ratings are displayed in Table 6.

**TABLE 6.** Means and Standard Deviations for Estimated Intelligences of Four Vignettes

	Feminine female		Masculine female		Feminine male		Masculine male	
Intelligence	*M*	*SD*	*M*	*SD*	*M*	*SD*	*M*	*SD*
General IQ	107.76	8.43	121.88	10.72	109.29	8.97	122.64	11.84
Verbal IQ	108.95	9.66	119.19	11.47	109.69	10.45	119.69	11.86
Maths/logical IQ	103.57	8.72	122.05	12.01	102.10	19.18	122.91	12.97

There were main effects of gender role (*F*(2,259) = 692.67, *p* < .001) and gender (*F*(2.259) = 12.55, *p* < .001) on general intelligence with masculine targets being rated higher than feminine and males higher than females. On verbal and mathematical intelligences there was a main effect of gender role with higher scores awarded to masculine targets. The results of verbal and mathematical IQ ratings support *H2a* and *H2b*. However, in relation to general IQ estimates only the former hypothesis was supported. Next, two series of the same analyses with participants’ gender and gender role as between-subjects factors were conducted. Neither participants’ gender nor gender role effected their ratings of targets’ general or mathematical intelligences which was contrary to what had been hypothesised (*H2d*). On verbal intelligence, however, there was an interaction of targets’ gender and gender role with participants’ gender (*F*(2,259) = 5.81, *p* < .05) and targets’ gender and gender role with participants’ gender role (*F*(2.259) = 2.94, *p* < .05). The mean for masculine males was 119.69 while that of feminine females was 108.95.

Estimating feminine vignettes, female participants gave higher estimates to male targets, whilst no significant gender differences were found in estimates of female targets. However, when masculine targets’ intelligence was estimated, male and female participants attributed the same scores to female target but male participants gave the male target higher and women lower scores. In regards to gender role differences, rating feminine vignettes, feminine, masculine and androgynous participants awarded higher scores to the male target and only the undifferentiated participants saw female target as more verbally intelligent. When masculine vignettes were being rated, undifferentiated and masculine participants rated the male target higher, whereas feminine and androgynous participants awarded higher scores to the female target.

To compare ratings of mathematical and verbal intelligence for each subject four paired-samples *t*-tests were run. The results, shown in Table 7, revealed that regardless of their gender, feminine targets were judged to have higher verbal than mathematical intelligence, whereas in case of masculine targets the opposite was true. Thus *H2c* was supported.

**TABLE 7.** Comparisons of Verbal and Mathematical Intelligence Ratings for Four Targets (Paired Samples *t*-Tests)

Vignette	Mean	*SD*	*t*	*df*
Alice (feminine)	5.03	9.71	9.52***	336
Susan (masculine)	−2.82	9.98	−5.36***	359
Jonathan (feminine)	6.41	16.94	7.06***	347
Martin (masculine)	−3.06	10.73	−5.37***	353

*** *p* < .0001 (two tailed).

Positive values indicate higher verbal IQ ratings.

### Vignettes’ Personality Traits Rating

A MANOVA was then computed to compare masculinity/femininity ratings of male and female targets when the information about their high IQ was or was not provided. There was a significant effect of intelligence on both masculine (*F* = 8.64, *p* < .001) and feminine (*F* = 12.58, *p* < .001) ratings. The intelligent female were rated as significantly more masculine than less intelligent female (*p* < .001) and less intelligent male (*p* < .01) and equally masculine as intelligent male (*p* = 1). The less intelligent female and less intelligent male were seen as equally masculine (*p* = 1). The effect of high intelligence on femininity was somewhat different: The intelligent female was seen as less feminine than the less intelligent female (*p* < .01) but more feminine than the intelligent male (*p* < .05); there were no differences in femininity ratings of the intelligent female and less intelligent male (*p* = 1). The less intelligent female was rated more feminine than all other targets (intelligent female, *p* < .01; intelligent male *p* < .001; less intelligent male *p* < .05) and intelligent male as less feminine than all other targets (intelligent female *p* < .05, less intelligent female *p* < .001; less intelligent male *p* < .01)

Finally, a MANOVA on the remaining four questions was carried out to compare the effect of target's sex and intelligence (four description's versions) on how likeable, attractive, masculine/feminine and likely to be single they seem. There was a difference between the four descriptions only on their apparent likeability (*F* = 14.47, *p* < .001). Participants thought that the intelligent male was less likeable than all other targets (*M* = 3.4, *p* < .001). The less intelligent male appeared to be most likeable (*M* = 4.06): “He” was liked more than the intelligent woman (*M* = 3.81, *p* < .0.5) but there was no difference between him and the less intelligent female (*M* = 3.99). The two female targets also did not differ significantly in their likeability.

## Discussion

The results indicate that gender role differences were stronger in self-estimates of “personal” than cognitive abilities, where gender differences were more salient. Also, male gender was predominantly positively related to perceived cognitive/intellectual abilities, whereas female was related to rated “soft,” personal skills. The effect of gender was strongest on mathematical, emotional, and spatial intelligences, but in the case of verbal, social, and practical, only gender role was important. There was a consistent, self-enhancing effect of masculinity even in such traditionally feminine areas as social and verbal abilities. Its effect was positive (although not significant), which was also true of emotional intelligence. Femininity was positively related only to self-estimated personal skills, whereas its relationship with general, verbal, and mathematical intelligence self-estimates was negative (but not significantly so).

Altogether, it seems then that it is not a “self-derogatory or self-effacing” effect of adopting feminine cultural stereotypes but rather self-enhancing effect of adopting the masculine ones that affects self-perception of intelligence. That is, this study, suggests more evidence of male hubris when it comes to evaluating abilities than female humility. This possibly explains why there was no effect of gender role on females in Rammstedt and Rammsayer's (2002) study, who did not examine cross-gender-typed individuals and thus the effects of masculinity on females. The results of the current study, however, show such an effect on verbal and social intelligences. Similarly, not distinguishing between undifferentiated and androgynous groups was probably the reason for the lack of gender role effects on personal factors in Rammstedt and Rammsayer's (2000, 2001, 2002) research. The only differences present in self-estimated personal skills in the present study involved undifferentiated females (rating themselves lower than all other females on social and than androgynous females on emotional intelligence) and androgynous males (who gave higher self-estimates than their masculine and undifferentiated counterparts).

The gender role effect on the differences between estimates for self and partner was much less pronounced, while the differences between masculine and feminine groups were in all cases high. Moreover, there was a consistent pattern of higher masculine subjects’ self-estimates on cognitive and lower on personal intelligences in relation to partner's estimates, as well as the self-enhancing effect of masculinity and self-derogatory effect of femininity.

The results for self and partner's estimates of general IQ reveal possibly important methodological implications. Although neither sex nor gender role affects either of these ratings, this was due to the statistical test (two-way ANOVA) chosen. However, when the effect of gender was analyzed separately (one-way ANOVA), the F value increased twofold and was significant at the α = .01 level. A problem for this area is that gender and gender role (according to Bem's categorical scheme) are inevitably confounded.

The results revealed that only the “traditional” cognitive abilities were associated with general intelligence (Furnham, 2001). It shows that despite high popularity of the concepts of emotional and interpersonal “intelligences,” they are still perceived as essentially a “social skill,” not related to general IQ. Yet, the presence of verbal intelligence, traditionally regarded as more feminine, among the two strongest predictors of overall IQ may seem to contradict this idea. However, it should be noted that although meta-analyses of verbal intelligence (Hyde, 1981) showed slight female advantage (*d* = −0.11), this is not reflected in people's self-estimates, which favor men (*d* = 0.32 in the present study). This opposite effect could be a result of an overriding belief that males are superior to females in cognitive abilities, which affects ratings of individual cognitive skills.

The results of vignettes’ IQ ratings revealed clear associations between intelligence and gender-related personality traits, showing that intelligence is indeed part of gender role, with higher IQ associated with masculinity. Differences in vignettes’ gender-related personality traits’ ratings show that intelligence is regarded as a more masculine than feminine attribute. The information about high intelligence overrode the effect of targets’ gender on the masculinity dimension, which was only partly true in relation to femininity. This asymmetry could possibly be explained by the fact that as masculinity and femininity are separate dimensions, masculine characteristic (in this case high IQ) has the largest effect on other masculine traits and smaller on feminine.

The masculine targets were judged as significantly more intelligent compared to the feminine ones. Here again the gender-role characteristics of the targets overrode the effect of their gender. Although there was a significant effect of gender on general IQ estimates, the differences in scores were less than one IQ point which makes only marginal difference. It was also revealed that verbal intelligence is perceived as more feminine than masculine as the feminine targets were rated more verbally, than mathematically, intelligent whereas the opposite was true for masculine vignettes. For future research, however, it seems advisable to separate the occupation's and personality traits’ effects on intelligence estimates and examine the impact of masculine vs. feminine professions that are closer in social hierarchy (Furnham & Rawles, 1995).

It should be stressed that associations between intelligence and personality traits’ ratings were not limited to gender-typed participants. It suggests that intelligence-related gender stereotypes are relatively common and shared by males and females, although their effects on individuals’ self-evaluation of abilities is moderated by their gender role. Gender role partly explains the disparities between self-estimated and psychometrically measured intelligences. When effect sizes for gender differences are computed with the effects of gender role being co-varied, they are closer to the *d*s for actual abilities (Hyde et al., 1990; Hyde & Linn, 1988; Voyer et al., 1995) than when gender role is not controlled for. However, that does not completely account for the “male hubris, female humility” effect as disparities still exist especially on verbal IQ.

One issue this study could not address was the role of national and/or ethnic culture in the formation and maintenance of these beliefs. Although now over 30 studies done in different countries have demonstrated the consistence of the sex differences in self-rated intelligence, the same has not been done for gender-role which has been shown to be more culturally determined (Szymanowicz & Furnham, 2011a, 2011b). Hence the importance of cross-cultural replications such as the American study by Kaufman (2012) or the Spanish study by Perez, Gonzalez, & Beltran (2010).

There were various limitations of this study. The sample though adequate was small. Further it would have been desirable to search for a larger group of undifferentiated and androgynous of both sexes so that analyses could be done separately for males and females. It would have also been desirable to use other gender-role measures such as that of Lippa (2005) while validating the use of the rather dated BSRI in a different culture. Also we did not vary the order in which the various parts of the questionnaire were presented. It would also be desirable to have more vignettes to ensure the reliability of the findings.

There are practical implications for this study. Whilst the literature consistently show little or no sex difference in overall intelligence, the hubris-humility effect of sex differences still occurs across time, populations, and ratings. If self-beliefs shape behavior, this may be good for males in terms of self-enhancement processes but bad for females in terms of self-degradation processes. One implication is to spend more time with females who, following cultural demands for hubris, under-estimate their abilities and then self-fulfil their erroneous assumptions about their ability.

There are a number of interesting and important future directions for this research. First, it is always desirable to run psychometric IQ tests alongside self-estimates to see whether females are under-estimating, males over-estimating or both phenomena are occurring. Second, it would be interesting to make a study of outliers who give very high and very low scores, particularly those females who give high scores and males who give low scores, to see when and why they do this. Third, given that test feedback might change perceptions it seems important to study how, whether, and when giving people feedback on their actual intelligence changes their self-estimates and for how long that lasts.

This study confirmed and extended the rapidly growing research in this area. It showed males tended to rate their mathematical and spatial intelligence higher, and emotional intelligence lower than females. It showed that gender role plays a smaller part than gender with masculinity being seen to be positively related to practical, but negatively related to emotional intelligence. Cognitive abilities/intelligence are seen to be male and masculine and personal/social abilities/intelligence seen to be female and feminine. Females tend to rate their (male) partner as being better at mathematical and spatial intelligence than themselves, but their social and emotional intelligence higher than their partners, while males do the exact reverse. The more masculine others are seen to be the more they are seen to have cognitive abilities and less personal/social abilities.

## AUTHOR NOTES

**Agata Szymanowicz** is affiliated with YSC, London. **Adrian Furnham** is affiliated with the Department of Psychology, University College London.
